# Review of *Phanoperla* (Plecoptera, Perlidae) from China

**DOI:** 10.3897/zookeys.1052.64060

**Published:** 2021-07-30

**Authors:** Raorao Mo, Ying Wang, Guoquan Wang, Weihai Li

**Affiliations:** 1 Guangxi key laboratory of Agric-Environment and Agric-Products Safety and National Demonstration Center for Experimental Plant Science Education, Agricultural College, Guangxi University, Nanning, Guangxi 530004, China Guangxi University Nanning China; 2 Department of Plant Protection, Henan Institute of Science and Technology, Xinxiang, Henan 453003, China Henan Institute of Science and Technology Xinxiang China

**Keywords:** *
Neoperlops binodosa
*, new combination, new species, new synonym, *Phanoperlacheni* sp. nov., *
P. hainana
*, *
P. pallipennis
*

## Abstract

Three Chinese species of the genus *Phanoperla* are reviewed. A new species, *P.cheni***sp. nov.**, is proposed from Guangdong, southern China, and compared with related taxa. *Neoperlopsbinodosa* Wu, 1973 is confirmed from Hainan Province of China on the basis of re-examination of types from the island, but it is transferred to the genus *Phanoperla* and is placed as a synonym of *P.pallipennis* (Banks, 1938). A note on the distribution of the genus *Phanoperla* is also given.

## Introduction

The perlid genus *Phanoperla* (subfamily Perlinae) was erected by Banks (1938) and is mainly distributed in the Oriental Region, with 52 species worldwide (DeWalt et al. 2021). In China, the first description of a *Phanoperla* species was based on specimens from Hainan Province by Banks (1938). After nearly a century, Li and Qin (2016) described *P.hainana* Li & Qin, 2016, the second Chinese species of the genus, also from Hainan. And recently, *Phanoperlahuanghuye* Chen, 2020 was shown to be a member of *Neoperlops* (Mo et al. 2020). During a collecting tour in the Nanling Mountains in September 2020, additional important material was collected from Chebaling National Natural Reserve of northern Guangdong Province, China, and it is described as new: *P.cheni* sp. nov. In addition, *Neoperlopsbinodosa* Wu, 1973 is herein considered a member of the genus *Phanoperla* and placed in the synonymy of *P.pallipennis* (Banks, 1938). In the present paper, a review of Chinese *Phanoperla* and a distribution map of the genus *Phanoperla* are presented.

## Materials and methods

The holotype of *Phanoperlacheni* sp. nov. was collected using a sweep net and is deposited in the Henan Institute of Science and Technology, Xinxiang (**HIST**). Types of *Neoperlopsbinodosa* Wu, 1973 are deposited in the National Zoological Museum of China, Institute of Zoology, Chinese Academy of Sciences, Beijing (**IZCAS**). The studied materials are stored in 75% ethanol. The holotype of *Phanoperlacheni* sp. nov. was examined with the aid of an Olympus SZ61 dissecting microscope, and color photographs were made with Keyence VHX-S650E and Leica M420 microscopes. The color photographs of *Neoperlopsbinodosa* Wu, 1973 were made with a Leica C camera with a lens of an Olympus SZX7 microscope in the IZCAS. Terminalia were removed from the abdomen and soaked in 10% NaOH. The aedeagus was everted using the cold maceration technique of Zwick (1983). Morphological terminology primarily follows that of Zwick (1982). The map (Fig. 7) was prepared using a base map downloaded from the standard map service of the online government service platform of the Ministry of Natural Resources, People’s Republic of China (http://bzdt.ch.mnr.gov.cn/; map number GS(2016)2938).

## Results and discussion

### Phanoperla cheni sp. nov.

Taxon classificationAnimaliaPlecopteraPerlidae

D4886379-57C4-5175-A9E8-B986B62F00D0

http://zoobank.org/406C9A78-A006-430B-A9F0-78BB63C95029

**Figure 1.**
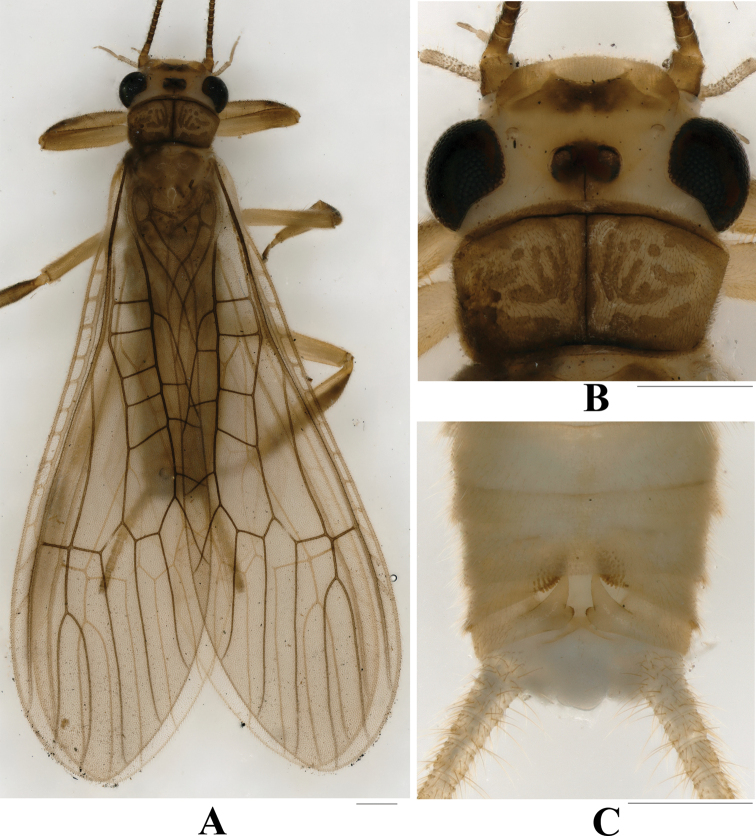
*Phanoperlacheni* sp. nov. (male) **A** adult habitus, dorsal view **B** head and pronotum, dorsal view **C** terminalia, dorsal view. Scale bars: 0.50 mm.

**Figure 2.**
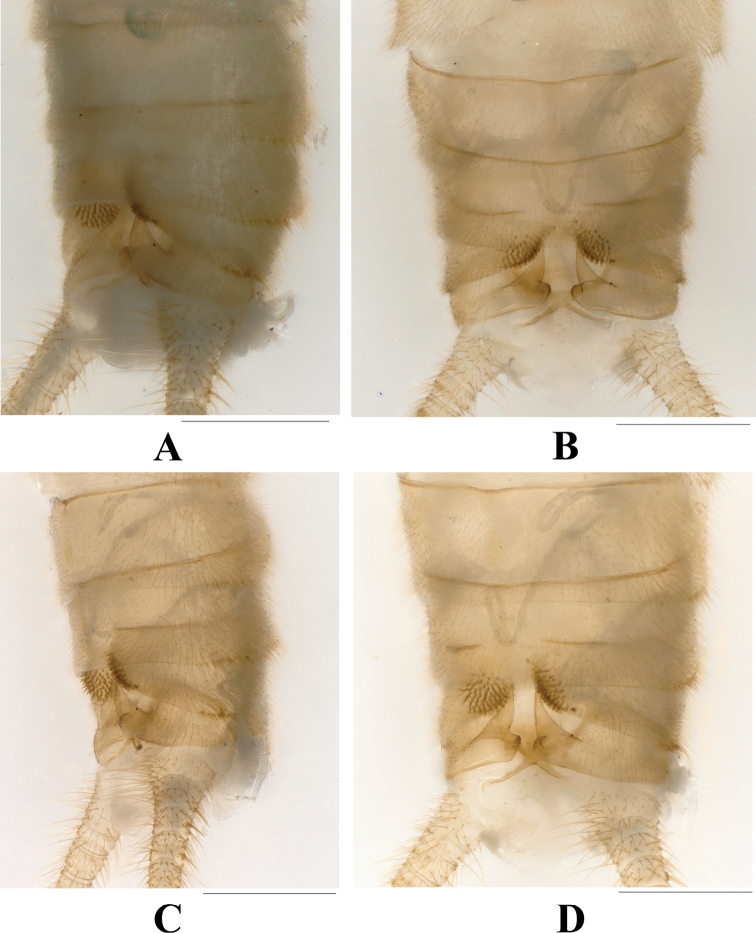
*Phanoperlacheni* sp. nov. (male) **A** terminalia, oblique dorsal view **B** terminalia after being cleared, dorsal view **C** terminalia after being cleared, lateral view **D** terminalia after being cleared, oblique dorsal view. Scale bars: 0.50 mm.

**Figure 3.**
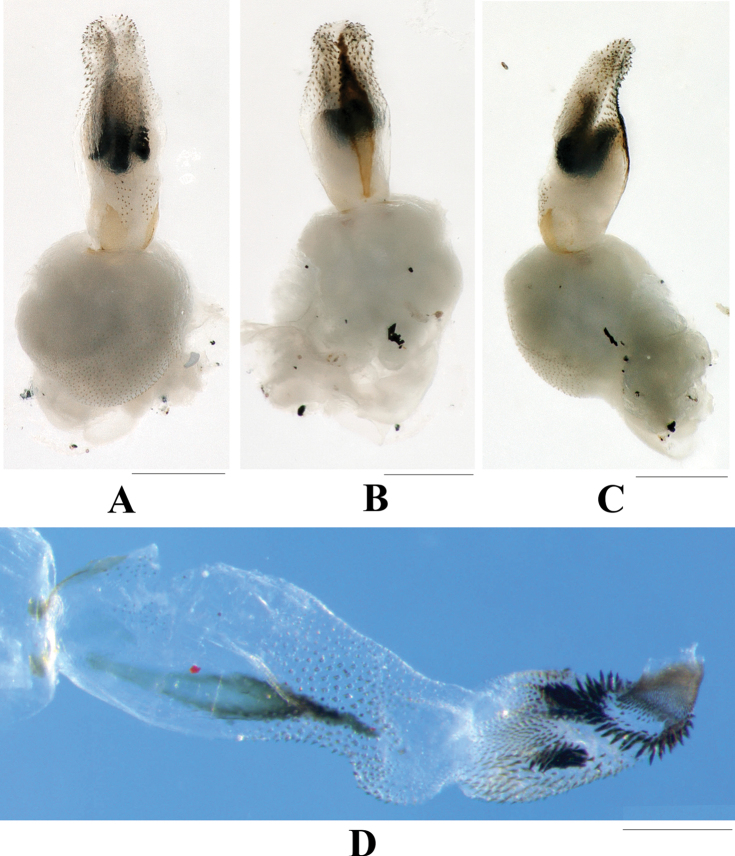
*Phanoperlacheni* sp. nov. (male) **A** aedeagus before eversion, ventral view **B** aedeagus before eversion, dorsal view **C** aedeagus before eversion, lateral view **D** aedeagus, ventral view. Scale bars: 0.25 mm.

**Figure 4.**
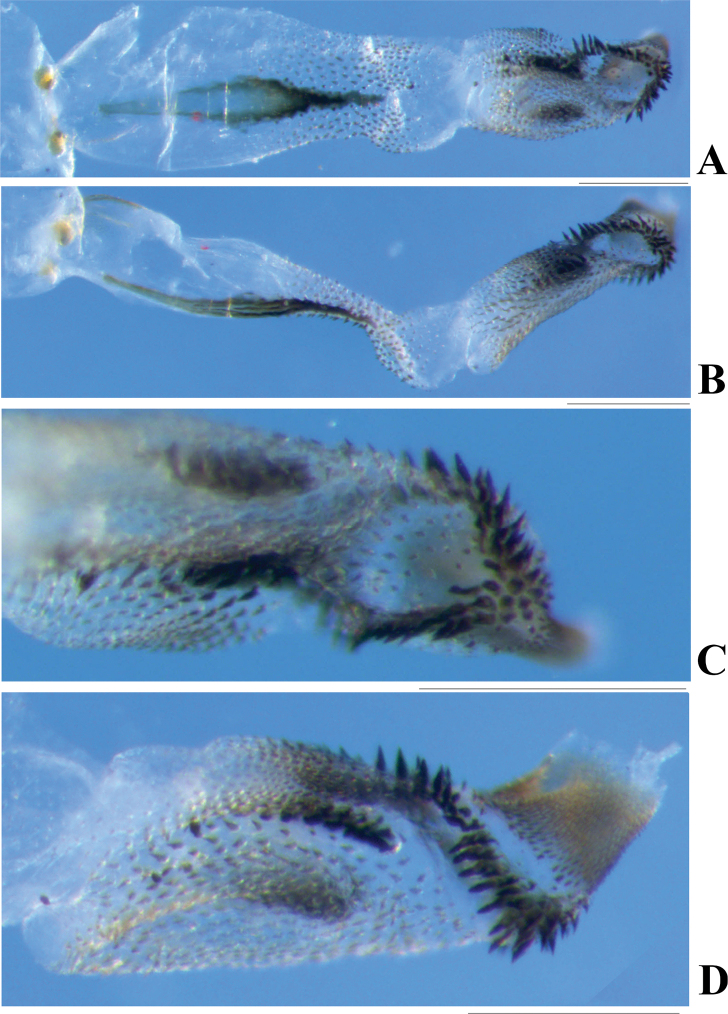
*Phanoperlacheni* sp. nov. (male) **A** aedeagus, dorsal view **B** aedeagus, lateral view **C** apical part of aedeagus, dorsal view **D** apical part of aedeagus, lateral view. Scale bars: 0.20 mm.

Figures 1 , 2 , 3 , 4

#### Adult habitus.

General body color yellowish brown. Biocellate, black rings around ocelli nearly connected, with a small brown marking covering ocelli. Head pale, with an M-shaped brown marking on frons; antenna brown, flagellum darker, and palpi brown; compound eyes black, large and bulging; head wider than pronotum. Pronotum rectangular, corners round with distinct brown rugosities (Fig. 1A, B). All legs brown, femora paler; wing membrane pale, veins brown; Rs two-branched, the angle Cu2 arising from Cu1 typical of the genus. Abdominal segments generally brownish.

**Male** (Figs 1–4). Forewing length ca 8.0 mm; hindwing length ca 6.7 mm. Sternum 5–7 with distinct medial ventral brush. Tergum VIII with posterior margin barely produced and sclerotized without sensilla basiconica. Tergum IX with two lateral groups of sensilla basiconica (Figs 1C, 2). Hemitergal processes of tergum X relatively stout, strongly sclerotized, the sharp tip curved outward.

**Aedeagus** (Figs 3, 4). Aedeagal envelope membranous with a large ventral patch of spinules. Aedeagal tube short, S-shaped in lateral aspect and dorsally expanded at apex: dorsal sclerite slender, band-shaped; basal surface with two ventrolateral groups of tiny spines; apical half fully covered with stout spines. Aedeagal sac as long as tube, curved ventrad, mostly covered with brown spines, but base bare: a pair of lateral black spine patches located in ventral surface of median half; apical half with a scarf-shaped band of larger black spines; apical part tubular, fully armed with tiny golden-brown spines.

**Female.** Unknown.

#### Type material.

***Holotype***: male (HIST), China: Guangdong Province, Shaoguan City, Shixing County, Chebaling National Natural Reserve, 24.72°N, 114.26°E, 327.4 m alt., 2020.IX.13, Chen Xulong leg.

#### Etymology.

The patronym honors the collector of the holotype; a noun (name) in genitive case.

#### Distribution.

China (Guangdong Province). The new species is known only from the Chebaling National Natural Reserve of Guangdong, which is in the Nanling Mountains. The Reserve has a subtropical monsoon climate, with heat and abundant rainfall throughout the year.

#### Remarks.

The male genitalia and aedeagal tube of the new species is similar to that of *P.wieng* Sivec & Stark, 2010 from Phrae Province of Thailand. However, in that species, the apical part of aedeagal tube is less expanded dorsally (compare Figs 3D, 4A, B with Sivec and Stark 2010: fig. 39–41). In addition, the aedeagal sac of *P.wieng* bears a dorsomedian hump and a pair of dorsobasal lobes, which are lacking in the straight aedeagal sac of the new species. Both species bear a similar spine arrangement of the aedeagal sac, but in the new species, a pair of small patches of black spines are present on the ventrolateral surface of the median half of the aedeagal sac, while the spine patches are lacking in *P.wieng*. The new species is also similar to *P.vietnamensis* Zwick, 1986 in its male terminalia, but their aedeagi are obviously different (compare Zwick 1986: figs 2, 3 with Figs 3, 4). In addition, the new species lacks two irregular rows of sensilla on tergum 9.

### Phanoperla hainana

Taxon classificationAnimaliaPlecopteraPerlidae

Li & Qin, 2016

1C8AA7F6-DDF7-5ED0-8886-C9524C310404

Phanoperla hainana Li & Qin, 2016: 193.

#### Distribution.

China (Hainan Province).

#### Remarks.

This species is a member of the *Phanoperlapallipennis* species group (Zwick 1982) and was described on the basis of the male holotype from Mount Limushan, which is located in Qiongzhong County, central Hainan Province (Li and Qin 2016). This species can be distinguished from all other Chinese species of *Phanoperla* by the unique head pattern and different aedeagus.

### Phanoperla pallipennis

Taxon classificationAnimaliaPlecopteraPerlidae

(Banks, 1938)

216FAB64-3121-5335-A91E-7C108BFD2ED2

Figures 5 , 6

Neoperla (Phanoperla) pallipennis Banks, 1938: 222; Illies 1966: 506.
Phanoperla pallipennis : Zwick 1982: 102; Du et al. 1999: 63; Li and Qin 2016: 193; Yang and Li 2018: 44.
Neoperlops binodosa Wu, 1973: 109. syn. nov.
Neoperla binodosa : Du et al. 1999: 63; Chen and Du 2016: 244; Yang and Li 2018: 44.

#### Type material

(*Neoperlopsbinodosa* Wu, 1973). 1 male and 1 female (with holotype and allotype labels) (IZCAS), China: Guangdong, Hainan, Yinggen (Hainan Province, Qiongzhong Li and Miao Autonomous County, Yinggen Town, 19.03°N, 109.83°E), 200 m alt., 1960.VII.5, Li Changqing (type no. 1466477–1466478) leg. Paratypes: 1 female (IZCAS), same data as for preceding (type no. 1466473); 1 male (IZCAS), same locality as for preceding, 1960.V.4, Li Suofu (type no. 1466474; 1960.VII.5, Li Changqing leg. in Wu 1973) leg.; 2 males (IZCAS), same locality as for preceding, 1960.V.10, Li Suofu (type no. 1466475–1466476; 1960.VII.5, Li Changqing leg. in Wu 1973) leg.; 2 males (IZCAS), same locality as for preceding, 1960.V.4, Li Changqing (type no. 1466480–1466481; 1960.VII.5 in Wu 1973) leg.; 1 male (IZCAS), Guangdong, Hainan, Tongshen (Hainan Province, Wuzhishan City, Tongshen Town, 18.76°N, 109.51°E), 350 m alt., 1960.III.25, Zhang Xuezhong (type no. 1466479) leg. Among these, one male and one female paratypes from Hainan, and a male from Yunnan was not found for checking.

#### Distribution.

Confirmed from Hainan province, but possibly also distributed in Yunnan province, as originally indicated.

#### Diagnosis and remarks.

**Figure 5.**
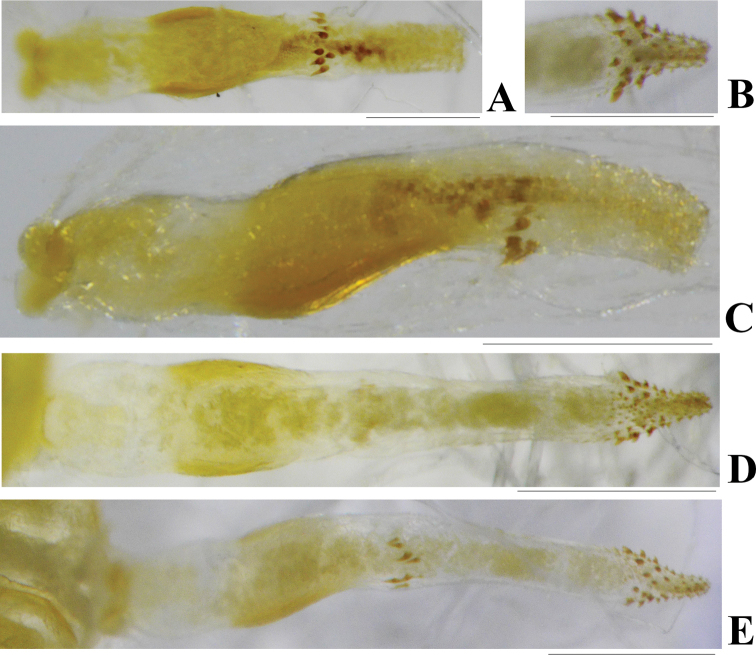
*Phanoperlapallipennis* (Banks, 1938) (male **A, C** holotype of *Neoperlopsbinodosa* Wu, 1973 **B, D, E** paratype of *Neoperlopsbinodosa* Wu, 1973) **A** aedeagus before eversion, dorsal view **B** apical part of aedeagus, dorsal view **C** aedeagus before eversion, lateral view **D** aedeagus, ventral view **E** aedeagus, lateral view. Scale bars: 0.20 mm.

**Figure 6.**
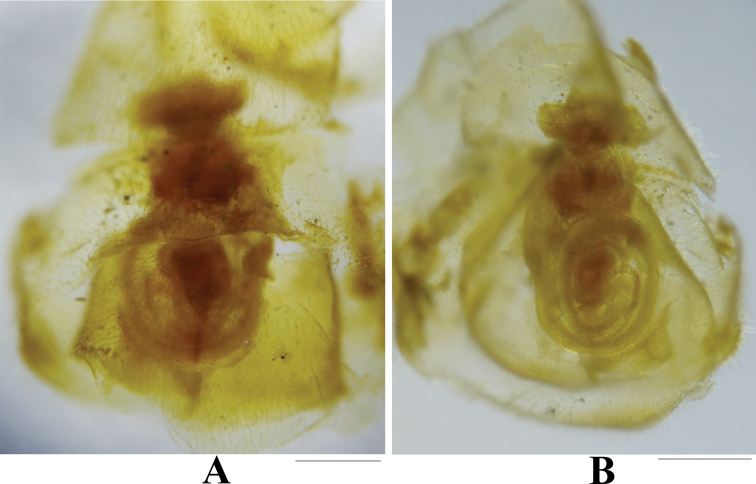
*Neoperla* sp. (female; allotype of *Neoperlopsbinodosa* Wu, 1973) **A** terminalia, ventral view **B** vagina, dorsal view. Scale bars: 0.20 mm.

*Neoperlopsbinodosa* Wu, 1973 was transferred to *Neoperla* by Du et al. (1999), but its status is still questionable because of the unknown male aedeagus (Du et al. 1999; Yang and Li 2018). We transfer *N.binodosa* to *Phanoperla* and synonymize it with *P.pallipennis* (Banks, 1938) on the basis of the identical male terminalia and main aedeagal armatures (compare Zwick 1982: fig. 10a, d with Fig. 5). The subgenital plate of female specimens agrees well with the original description and figures by Wu (1973), being scarcely produced and without an apical notch (Fig. 6), which differs from the female paratypes of *P.pallipennis*. Therefore, we propose that female specimens in IZCAS may be not conspecific with male specimens and may even not belong to the genus *Phanoperla*. The female subgenital plate of *Phanoperla* is usually slightly produced and typically bilobed, with a sausage-like or balloon-like spermatheca (Sivec et al. 1988; Cao and Bae 2009). Consequently, we boldly postulate that this female is *Neoperla* sp., because of the barely produced subgenital plate and a coiled spermatheca which is common in *Neoperla*. Unfortunately, the female specimens in the IZCAS are badly damaged and should be confirmed by new studies.

## Concluding remarks

**Figure 7.**
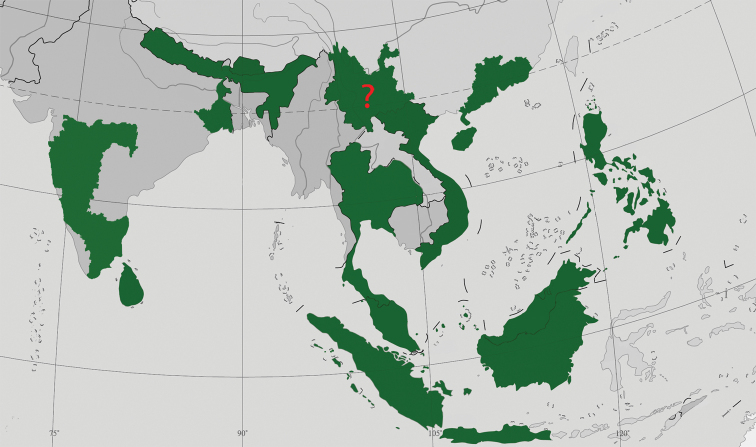
Distribution of *Phanoperla* Banks, 1938.

The genus *Phanoperla* currently consists of 52 valid species and is restricted to the Oriental Region (Fig. 7), mostly occurring in the Indonesian archipelago and Thailand (DeWalt et al. 2021). The wide distribution of *Phanoperla* in the Oriental Region, which covers nearly two-thirds of the region, suggests that more species will be found there, even in areas where the genus is unreported, such as the western of part the Himalayan region, Bangladesh, Myanmar, Laos, and southwest and south China.

The major references dealing with Chinese *Phanoperla* are Banks (1938) and Li and Qin (2016); these include only with two species: *P.hainana* Li & Qin, 2016 and *P.pallipennis* (Banks, 1938), both occurring in Hainan Province of southern China. *Neoperlopshuanghuye* (Chen, 2020), from Fujian Province, was placed in *Phanoperla* in a previous study but was re-evaluated to belong to *Neoperlops* on the basis of the male and female terminalia, aedeagus, and eggs (Mo et al. 2020). In this study, the third *Phanoperla* species from China, *P.cheni* sp. nov., is described from Guangdong Province, which faces Hainan across the South China Sea. And *Neoperlopsbinodosa* Wu, 1973, a synonym of *P.pallipennis*, was known from Hainan and Yunnan provinces (Wu 1973), but as the Yunnan paratype was not found and the allotype proved to belong to another species, presence in Yunnan must still be confirmed.

## Supplementary Material

XML Treatment for Phanoperla cheni

XML Treatment for Phanoperla hainana

XML Treatment for Phanoperla pallipennis
